# The baculum affects paternity success of first but not second males in house mouse sperm competition

**DOI:** 10.1186/s12862-021-01887-6

**Published:** 2021-08-12

**Authors:** Lennart Winkler, Anna K. Lindholm, Steven A. Ramm, Andreas Sutter

**Affiliations:** 1 Department of Evolutionary Biology, Bielefeld University, Konsequenz 45, 33615 Bielefeld, Germany; 2 Department of Evolutionary Biology and Environmental Studies, University of Zurich, Winterthurerstrasse 190, 8057 Zurich, Switzerland; 3 School of Biological Sciences, Norwich Research Park, University of East Anglia, Norwich, NR4 7TJ UK; 4 Present Address: Applied Zoology, Technical University Dresden, Zellescher Weg 20b, 01062 Dresden, Germany

**Keywords:** Genital evolution, Sexual selection, Fertilization success, Mating behavior, Geometric morphometrics, Copulatory plugs

## Abstract

The vast variation observed in genital morphology is a longstanding puzzle in evolutionary biology. Studies showing that the morphology of the mammalian baculum (penis bone) can covary with a male’s paternity success indicate a potential impact of baculum morphology on male fitness, likely through influencing sperm competition outcomes. We therefore measured the size (measurements of length and width) and shape (geometric morphometric measurements) of the bacula of male house mice used in previously published sperm competition experiments, in which two males mated successively with the same female in staged matings. This enabled us to correlate baculum morphology with sperm competition success, incorporating potential explanatory variables related to copulatory plugs, male mating behavior and a selfish genetic element that influences sperm motility. We found that a wider baculum shaft increased a male’s paternity share when mating first, but not when mating second with a multiply-mating female. Geometric morphometric shape measurements were not clearly associated with fertilization success for either male. We found limited evidence that the effect of baculum morphology on male fertilization success was altered by experimental removal of the copulatory plug. Furthermore, neither genetic differences in sperm motility, nor covariation with male mating behavior mediated the effect of baculum morphology on male fertilization success. Taken together with previous findings, the mating-order effects we found here suggest that baculum-mediated stimulation by the first male might be particularly important for fertilization.

**Supplementary Information:**

The online version contains supplementary material available at 10.1186/s12862-021-01887-6.

## Background

Extensive variation in genital morphology in animals with internal fertilization is found among many species, even closely related ones [1–4]. Hypotheses for drivers of genital evolution include female choice, male-male competition, sexual conflict, natural selection—for example via species-isolating lock-and-key mechanisms—or pleiotropic effects [1, 3, 5–8].

The baculum (*os penis*) is a bone located within the penis, found across several mammalian taxa including many rodent species [9, 10]. It exhibits diverse morphology [9–12] that is thought to be driven by sexual selection [2]. Besides mere size variation, baculum shape varies greatly between species, from very simple to elaborate bones equipped with spikes and spoon-like structures [9, 13]. Its diversity and its potential key role in male reproductive success make the baculum an interesting subject for studying the evolution of genital morphology.

Several non-mutually exclusive functions of the baculum have been suggested. The baculum might serve as mechanical support for the penis to overcome vaginal resistance [14], or might protect the urethra from compression during copulation [15]. Further, the baculum may be important for female stimulation, as it enters the vagina during copulation [16], and facilitated intromission might increase male reproductive success [17, 18]. For example in mice, vaginal stimulation before ejaculation increases litter size [19], and vaginal distension is important for the success of artificial insemination [20]. A phylogenetic study of male rodents found a positive correlation between baculum length and relative testis size, supporting an association with postcopulatory sexual selection [21], but this correlation was not found in carnivores and primates [22]. The fact that the baculum is not a homologous structure but has been gained and lost several times [12] may explain these contradictory results [21, 22]. Besides length, baculum width may also be important, as greater width is associated with male dominance [23] and male reproductive success [24], although it remains unclear precisely why. Finally, in species where males deposit copulatory plugs inside the female genital tract during copulation, baculum morphology might also influence their placement and/or removal. In rodents, plugs appear important to ensure sperm transport [25], embryo implantation [26] and to delay ejaculation of rival males [27, 28], thereby playing an important role in rodent sperm competition [10, 27, 29, 30]. The baculum could influence copulatory plug functionality in two possible ways: first by enabling the plug to be placed appropriately, and second by easing the removal of a rival male’s plug from a previously mated female.

One intensely studied model organism for baculum evolution is the house mouse (*Mus musculus* [10, 24, 31–33], whose polyandrous mating system [34, 35] is a potential driver for sexual selection on the baculum. Despite this relative wealth of research, the precise function of the house mouse baculum remains in many ways unexplored [10, 36]. Although recent evidence suggests that the baculum plays a role in stimulation of the female [37], many hypotheses on the function of the baculum have not been tested yet.

Stockley et al. [24] demonstrated that the baculum width of male house mice was associated with paternity success in semi-natural enclosures, whereas length was not. Using geometric morphometrics, Simmons and Firman [32] found accordingly that the baculum of male house mice was relatively wider in wild populations with higher levels of sperm competition. Additionally, they reported evolved divergence in baculum width after 27 generations of experimentally including or eliminating postcopulatory sexual selection, by allowing polyandry vs enforcing monandry [32]. Their results demonstrate that evolution by means of sexual selection can drive differences in baculum morphology.

Here, our main aims were to explicitly test the association between baculum morphology and male fertilization success under sperm competition, and to explore different hypotheses about potential underlying mechanisms. We measured baculum morphology of male house mice that had been used in staged sperm competition experiments [28, 38, 39]. These experiments had revealed that a meiotic driver reduced sperm competitiveness through its impact on sperm motility, particularly for first-to-mate males [27, 39], and that copulatory plugs deposited by first-to-mate males increase paternity success by delaying rival male ejaculation [28]. Using remaining variation in fertilization success, we here explore whether baculum morphology might be involved in (i) increasing efficiency in sperm transport, (ii) better positioning/facilitating removal of a copulatory plug, and/or (iii) enhancing copulatory performance of the male.

(i) *Can a particular baculum morphology aid individuals with low sperm motility to improve fertilization, supporting a role of baculum morphology in sperm transport?* To test this, we make use of genetic variation in sperm transport efficiency induced by whether a male is a carrier of the *t* haplotype. This meiotic driver is a selfish genetic element found at appreciable frequencies in natural house mouse populations [40, 41]. It is known to cause reduced sperm motility [42] but no reduction in sperm numbers or testis mass [38]. *t* haplotype carrier (+ /*t)* males are severely inferior in sperm competition against wildtype males (+ / +; [38, 39]). Crucially, + /*t* males are also not able to take advantage of first male sperm precedence that characterizes house mouse matings [38]. A comparison between the situations when two + /*t* males compete and when two + / + males compete can therefore test whether promoting sperm transport via baculum morphology is more important for + /*t* males, due to their impaired sperm motility. We hypothesize that if promoting sperm transport is an important function of the baculum, when two males of the same genotype compete, + /*t* males gain more benefits (i.e., fertilization success) from a superior baculum morphology than do wildtype males. We thus predict that the effect of baculum morphology on fertilization success is stronger in + /*t* vs + /*t* competition, as it may mitigate inferior sperm motility.
(ii) *Does baculum morphology matter for deposition and/or removal of copulatory plugs?* If certain baculum morphologies help to better place the copulatory plug, artificial plug removal after mating should eliminate any fertilization advantage for first-to-mate males with a baculum morphology favored in natural matings. If the baculum helps to remove the plug of rivals, the advantage of a certain morphology for second-to-mate males should only be seen when plugs are not experimentally removed. In previous laboratory experiments on postcopulatory effects of baculum morphology, copulatory plugs were always removed [32, 43]. We therefore compared associations between baculum morphology and paternity success in sperm competition experiments with and without plug removal.
(iii) *Does baculum morphology covary with male copulatory behavior?* Baculum morphology might influence male copulatory performance. For example, penis circumference and thus baculum width might influence the degree of stimulation during mating, and males with a wider baculum could express less vigorous copulatory behavior (i.e., less time spent in copulation as sufficient stimulation is reached faster), because they stimulate the female more efficiently. If there is plasticity in baculum-dependent mating behavior, we predict that baculum morphology correlates with copulatory behavior, more specifically that wider bacula correlate with shorter and fewer copulatory bouts.

Finally, in testing these hypotheses we also compare two previously used measures of baculum morphology. We do so by exploring the sensitivity of our analyses to relying either on direct baculum size measures (which we call size; e.g., [24]) or extending these to applying geometric morphometrics (which we call *shape*; e.g., [31, 32]). To our knowledge, the baculum has been studied using either direct size or morphometric shape measures, but never combining these approaches. We aim to investigate if both techniques yield similar results or if they measure different qualities of the baculum, which might explain why past studies employing different methods sometimes appear contradictory [31, 33].

## Results

### Males with a wider baculum gain higher paternity success

**Fig. 1:**
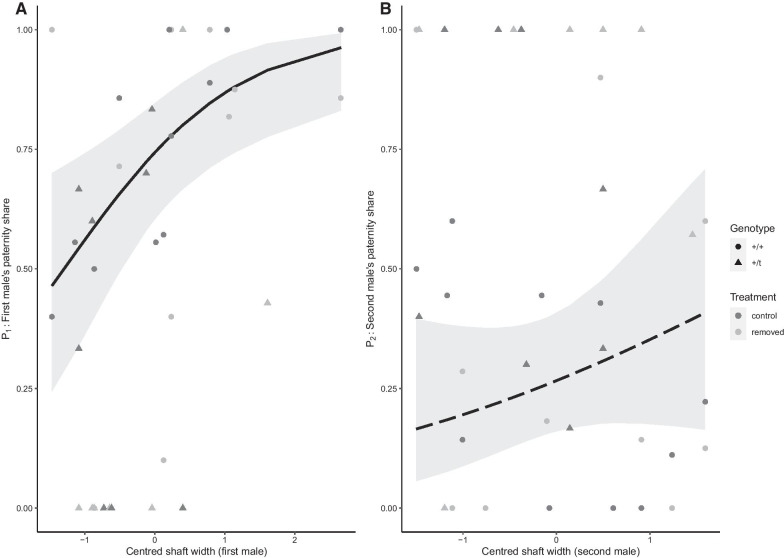
**A** Proportion of embryos sired by the first male (P_1_) plotted against shaft width of the first-to-mate male and **B** the proportion of embryos sired by the second male (P2) plotted against shaft width of the second-to-mate male. Baculum measures were standardized to a mean of 0 and a SD of 1. Regression lines represent predicted values (with 95% confidence intervals in grey) from beta-binomial GLMMs, with a dashed line indicating a non-significant effect

We first analyzed the effect of baculum morphology on fertilization success. In our full model, we found a significant positive effect of the first-to-mate male’s shaft width on the proportion of embryos sired (P_1_, proportion of embryos sired by the first-to-mate in competition over fertilization with a second male; adjusted p = 0.007; Fig. 1A, Table 1), while baculum base width (Additional file 1: Figure S6) and length had no significant effect on P_1_ (Table 1). In the reduced model identified by lowest AICc, both shaft width and base width were significantly positively correlated with P_1_ (see Additional file 1: Table S19). None of the morphometric (shape) measurements of first-to-mate males, and neither size nor shape of the baculum of second-to-mate males predicted paternity share (see Fig. 1B and Table 1).

**Table 1:** Results of beta-binomial mixed models for effects of baculum size (length, shaft width and base width) or morphometrics (relative warps 1 & 2 (RW)) on fertilization success of the first male relative to the second male (P_1_), including body mass and genotype as covariates

Model	Variable	Estimate	Std. error	z value	p-value	p-adj
Baculum size resid. df = 27	Intercept	1.38	0.43	3.19	0.001	0.007
	Length M1	0.17	0.38	0.44	0.660	0.724
	Shaft width M1	1.66	0.53	3.12	0.002	0.007
	Base width M1	0.32	0.28	1.11	0.267	0.367
	Length M2	− 0.53	0.37	− 1.42	0.154	0.243
	Shaft width M2	− 0.62	0.42	− 1.47	0.141	0.243
	Base width M2	− 0.10	0.28	− 0.35	0.7244	0.724
	Body mass M1	0.37	0.36	1.03	0.303	0.371
	Body mass M2	0.91	0.36	2.53	0.011	0.031
	Genotype M1 and M2 (+ /t)	− 2.40	0.58	4.15	< 0.001	< 0.001
	Plug removal (removed)	− 0.98	0.54863	− 1.796	0.07257	0.160
Baculum shape (morphometrics) resid. df = 25	Intercept	1.02	0.47	2.17	0.030	0.166
	log_10_ Centroid size M1	0.15	0.37	0.41	0.684	0.912
	RW1 M1	0.66	0.39	1.71	0.088	0.322
	RW2 M1	− 0.11	0.35	− 0.31	0.754	0.912
	log_10_ Centroid size M2	0.007	0.52	0.01	0.989	0.989
	RW1 M2	− 0.19	0.37	− 0.53	0.596	0.912
	RW2 M2	− 0.29	0.32	− 0.91	0.361	0.795
	Body mass M1	− 0.15	0.40	− 0.389	0.699	0.912
	Body mass M2	0.51	0.46	1.10	0.271	0.746
	Genotype M1 and M2 (+ /*t*)	− 2.77	0.83	− 3.35	< 0.001	0.009
	Plug removal (removed)	− 0.12	0.54	− 0.22	0.829	0.912

M1/M2 = first/second-to-mate male

As shown before [38], *t* genotype influenced sperm precedence patterns (Table 1). Furthermore, + /*t* and + / + males did not differ in baculum morphology (Additional file 1: Table S17). Importantly, there was no significant interaction between genotype and baculum measures (size or shape), either for first- or second-to-mate males (Additional file 1: Tables S5–S8), indicating that there was no specific baculum morphology that mitigated the negative effect of the *t* haplotype on paternity share.

### Optimal baculum morphology may change with experimental plug removal

**Fig. 2:**
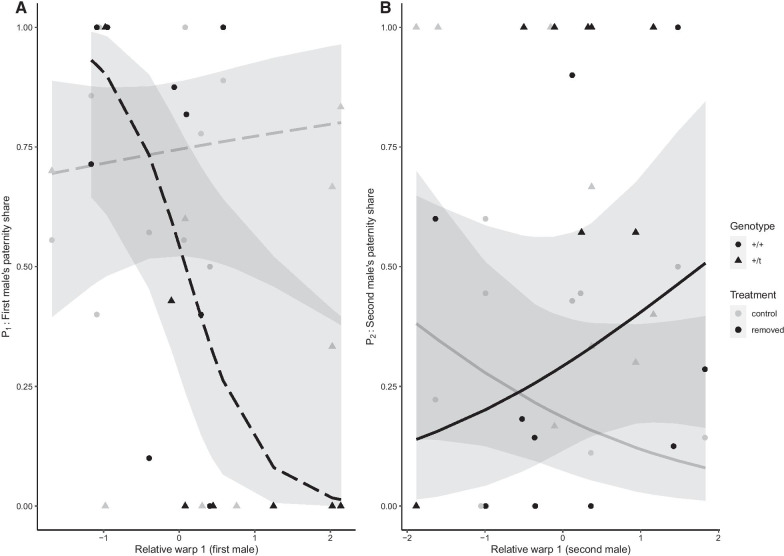
**A** Proportion of embryos sired by the first-to-mate male (P1) plotted against the relative warp 1 of the first-to-mate male. **B** Proportion of embryos sired by the second-to-mate male (P_2_) plotted against the relative warp 1 of the second-to-mate male. Baculum measures were standardized to a mean of 0 and a SD of 1. Regression lines represent predicted values with 95% confidence intervals from a beta-binomial GLMM shown separately for plug removal treatment (black) and control males (grey), with dashed lines indicating a non-significant interaction

The copulatory plug was experimentally removed after the first and second mating in some mating trials, a fact which we used here to test if baculum morphology might affect paternity outcomes via its effects on plug deposition and/or removal. Experimental removal of the copulatory plug did not influence how baculum morphology of first-to-mate males (size or shape) affected paternity, indicating no support for a role in plug deposition (Fig. 2A and Additional file 1: Tables S9 and S10). For second-to-mate males, there were no significant interactions with plug removal concerning size measurements (Additional file 1: Table S11). However, there was a significant interaction between plug removal treatment and RW1 (relative warp 1) of the second-to-mate male (adjusted p = 0.040; Table 2): when the plug of the first male was left intact, second-to-mate males with a smaller RW1 score (meaning a more ‘elongated’ base; Additional file 1: Figure S2) obtained a larger paternity share P_2_ (proportion of embryos sired by the second-to-mate male in competition over fertilization with the first-to-mate male). In contrast, when plugs were experimentally removed, there was a positive effect of larger RW1 on P_2_ (Fig. 2B). These results point towards a potential benefit of a more elongated (and thinner) baculum base for more efficient plug removal. However, individually neither of the slopes were significantly different from zero and the best model (AICc model selection) did not include any interactions (Additional file 1: Table S20).

**Table 2:** Results of a beta-binomial mixed model for effect of baculum morphometrics of the second male on his fertilization success relative to the first male (P_2_), including interactions with plug removal treatment, body mass of the second-to-mate male and genotype as covariates

Variable	Estimate	Std. error	z value	p-value	p-adj
Intercept	− 1.21	0.47	2.57	0.010	0.040
Centroid size	− 0.62	0.55	1.11	0.265	0.331
Relative warp 1	− 0.78	0.47	1.65	0.098	0.245
Relative warp 2	0.14	0.34	0.41	0.680	0.756
Interaction: Centroid size x Plug removal	1.33	0.90	1.48	0.139	0.277
Interaction: Relative warp 1 × Plug removal	1.89	0.75	2.52	0.012	0.040
Interaction: Relative warp 2 × Plug removal	0.79	0.59	1.34	0.180	0.284
Body mass	− 0.43	0.33	− 1.29	0.198	0.284
Genotype (+ /*t*)	2.44	0.64	3.78	0.0001	0.001
Plug removal	0.01	0.60	0.03	0.979	0.979

Resid. df = 30

### No association between baculum morphology and copulatory behavior

In addition to paternity success, we investigated a potential link between baculum morphology and copulatory behavior. Neither size nor shape covaried with copulatory behavior of either first- or second-to-mate males (Additional file 1: Tables S13–S16).

## Discussion

Among pairs of competing male house mice, we found that baculum shaft width of the first-to-mate male predicted paternity share, whereas baculum characteristics of the second male did not. Previous studies had implicated the house mouse baculum in differential paternity success [24], particularly through postcopulatory sexual selection [32, 43]. Here, we found limited support for hypotheses relating to the benefits of baculum width for sperm transport and copulatory plug deposition and removal. Instead, the mating order effects found here suggest that baculum-mediated stimulation by the first male might be particularly important for fertilization.

Our data provide further evidence that the effect of baculum morphology on male reproductive success is mediated through sperm competition, influencing paternity share when females mate with multiple males. Sperm competition risk in natural house mouse populations is likely to exert substantial selection on males [34, 35, 44, 45]. Simmons and Firman [32] previously provided evidence that postcopulatory sexual selection influences the evolution of baculum morphology using experimental evolution. The pattern was also reflected in variation found among natural populations that varied in relative testis size and the frequency of multiple paternity, suggesting that staged laboratory matings reproduce selective pressures in the wild at least to some extent. Our findings support the notion that wider bacula benefit males in sperm competition.

How exactly baculum morphology influences paternity outcomes is less clear. Here, shaft width appeared to be the most important aspect of baculum morphology for paternity outcome. In contrast to a previous study by Stockley et al. [24], we did not find a significant correlation between base width and paternity success, at least in our full models. Even in the reduced models which suggested a minor effect of base width, the effect of shaft width on fertilization success was stronger than that of base width, with barely overlapping standard errors (Additional file 1: Table S19). Overall, support for an effect of base width is weak in the present data. Indeed, Stockley et al. [24] also found a much stronger effect of shaft width than base width on paternity success. On a technical note, significant associations of base width and paternity success might be the result of a “winner’s curse” (i.e., overestimated effect sizes leading to false-positives; [46, 47]) due to the model selection approach partly used here and in Stockley et al. [24]. Nevertheless, as we used full models when assessing the overall fitness effect of the baculum (Table 1), the effect of shaft width on male fertilization success was not caused by such a “winner’s curse”.

We found no correlation between the geometric morphometric measurements and the proportion of embryos sired (Table 1), suggesting that baculum size is more important than shape in this context. However, centroid size also did not predict P_1_, so we can further infer that not overall size but rather shaft (and perhaps base) width specifically is most important. Unlike André et al. [43], who found an association between baculum shape and male paternity success, our primary shape variable, RW1, did not predict paternity despite describing base width. This might be because it also incorporates baculum length, which does not seem to influence male fertilization success. Nonetheless, it remains unclear if the absence of an effect of RW1 is due to subtle differences in the measurements, lower power of the morphometric measure, or the lack of an effect of base width. We encourage future studies to follow our approach of incorporating both direct and geometric morphometric measurements, since these data might capture different aspects of baculum morphology and might not be fully interchangeable. Previous studies focusing on either size [33] or shape [31] found contrasting results with respect to plasticity in baculum morphology induced by cues of competition [31, 33], but it is unclear whether this was due to differences in how morphology was assessed.

Even in a relatively well-studied species such as the house mouse, the precise function of the baculum remains elusive. In the following we discuss the three hypotheses that we addressed here. First, we investigated a potential link between sperm transport and the baculum’s role in fertilization success, by asking if the *t* haplotype influenced the relationship between baculum morphology and paternity share. As + /*t* males produce sperm with altered motility [42, 48], a stronger effect of a beneficial baculum morphology on fertilization in + /*t* vs + /*t* than in + / + vs + / + competition might have indicated that the baculum is important for efficient sperm transport. We found no evidence that + /*t* males obtain a different benefit of a certain baculum morphology than do + / + males, even though + /*t* males exhibit different sperm characteristics (early hyperactivation/differential motility [42, 48]).

Second, we asked if the copulatory plug mediated the effect of shaft width on paternity share, via an enhanced ability either to place own plugs or remove those previously deposited by rivals. Alternatively, baculum morphology might correlate with the morphology of the plug a male deposits (e.g., a wider baculum correlating with larger copulatory plugs). The copulatory plug appears to be important for ensuring male paternity against rivals and enhancing sperm flow [27, 30], and baculum morphology might play a role in supporting these functions. We found limited evidence that the copulatory plug influences how baculum morphology relates to male fertilization success. Plug removal did not alter the effect that base and shaft width of the baculum had on paternity outcome of the first male. Nevertheless, the experimental plug removal was performed by regularly checking for plugs every 1–1.5 h (see “Methods”). This introduces variation in the timing of the removal that might have obscured an effect of the plug and baculum morphology on paternity share (via enhanced sperm transport or delaying re-mating). Therefore, the absence of an interaction of plug removal and baculum morphology of the first-to-mate males has to be interpreted with the necessary caution. Nevertheless, we did find an interaction between plug removal treatment and the second-to-mate male’s RW1 on P_2_ (Table 2 and Fig. 2). When the copulatory plug was removed, second-to-mate males with a more ‘compressed’ baculum base (i.e., a wider but shorter base) were more successful. By contrast, in the control group where the copulatory plug was left intact, a more elongated baculum correlated with increased paternity. When copulatory plugs are removed, second males might benefit from a similar baculum morphology as first males. When plugs are left intact, optimal baculum morphology for second males might be an elongated rather than wide shape, which we hypothesise might enable them to partly by-pass copulatory plugs of first males to more easily remove them (akin to a crowbar). If optimal baculum morphology changes dependent on mating order, this might also help maintain variation in baculum morphology through balancing selection. However, more evidence is needed to corroborate such speculations. While genetic manipulation of baculum morphology of house mice seems to be currently impossible without any unwanted side effects on other bone structures (see [49]), manipulations of genital morphology are more feasible in other species. For example, in the red-sided garter snake experimental removal of the hemipene hook resulted in males depositing smaller copulatory plugs [50, 51] and in the seed beetle phenotypic engineering uncovered the importance of genital spines [52]. Making use of the range of species exhibiting copulatory plugs could further elucidate their possible evolutionary interplay with penis morphology.

Finally, we investigated a potential relationship between baculum morphology and male copulatory behavior. For example, if a wider baculum facilitates overcoming vaginal resistance, copulation durations might be shorter for males with a wider baculum. On the other hand, if the baculum aids sexual vigor [13], one might expect males with a beneficial morphology to be more active (e.g., performing more or longer copulatory bouts). Our data provide no evidence that baculum morphology relates to the copulatory behavior of males, and hence an influence on copulatory behavior cannot explain the association between baculum morphology and male fertilization success. We hypothesized that increased female stimulation by a beneficial baculum morphology might influence male copulatory behavior. Our data suggest that this is not the case. Nevertheless, we argue that this provides no strong evidence against the ‘stimulation hypothesis’, as optimal male mating duration is likely influenced by many factors. Together with high variation in behavior, this might conceal an effect of baculum morphology in our data. In addition, it is possible that there is no baculum-dependent plasticity in mating behavior, regardless of an influence of the baculum on female stimulation.

Overall, the tests we used to explore the different hypotheses on the function of the baculum are indirect and correlative. It is possible that chance, or differences in statistical power may have led to us finding an effect in first, but not second males. The power for detecting an effect of baculum morphology on male fitness could have been lower for the second-to-mate male, due to variation in the timing between the first and second mating. While the first male had control over the timing of his mating, the second male’s timing for mating was partly constrained by this. Nevertheless, our results provide the first evidence testing these hypotheses in combination in an integrative dataset. More direct experiments are needed to further illuminate the role of the house mouse baculum in sperm transport and copulatory plug removal. Additional experiments on the effect of the baculum on male fitness in sperm competition could illuminate this further.

We suggest, despite finding no effect of baculum morphology on male copulation behavior, that female stimulation is the most likely explanation for the effect that first male baculum morphology has on fertilization success. This is in line with a recent study finding that the effect of baculum shape on male mating success depended on the female’s breeding value for baculum shape, indicating coevolution between the sexes [43]. Taken together, these findings suggest that male genital morphology might be subject to cryptic female choice [53]. By means of Fisherian runaway selection [54],where a trait is exaggerated by a positive feedback of selection pressures on the male trait and the female preference for that trait, or ‘chase away’ selection ([55]; see ‘Red Queen hypothesis’; [56]), if females are stimulated beyond their optimum and evolve a higher stimulation threshold in response. Both ‘runaway’ and ‘chase away’ selection could provide an explanation to why house mice copulate for longer than appears necessary for sperm transfer alone [18, 19]. Males copulated to ejaculation in up to 60 bouts of intromittent behaviour, with an average of about 10 bouts [38], indicating that males often perform more copulatory bouts than needed simply for sperm transfer [57]. A recent comparative study of bacula reported that tip complexity was correlated with prolonged intromission, supporting a role of baculum morphology for female stimulation in other species [58]. If sufficient female stimulation had become harder to reach for males, males might evolve to both copulate for longer, and to provide more stimulation through wider bacula. Females might gain indirect benefits via ‘sexy sons’ [59] from selecting males with wide bacula. Nevertheless, we found no correlation between body weight or preputial gland weight (indicating dominance; [60]) and baculum shaft width (body weight: t_125_ = − 0.56, p = 0.576; preputial gland weight: t_126_ = 1.44, p = 0.151). Hence our data provide no evidence for the baculum being an honest signal of male quality, but female stimulation could still lead to a ‘sexy son’ advantage, as baculum morphology is heritable [37, 49]. As both competing males in the present experiment were brothers, high heritability would lead to low variation between competing males. This could lower the power of our design and hence pose a limitation to the present study. That we were nevertheless able to detect effects suggests overall high variability in baculum morphology in house mice. This is in line with previous findings that multiple genes control baculum morphology [37, 49].

An altogether different hypothesis for why males with wider bacula gain higher paternity success is that these males have superior sperm or seminal fluid quality or quantity. However, this hypothesis cannot explain that the effect of the baculum on paternity appears to be absent in the second-to-mate males, without assuming that sperm or seminal fluid quality are more important for first-to-mate males. In addition, controlling for testes- and seminal vesicle weight did not significantly alter the conclusions drawn from the model (Additional file 1: Table S18). Overall, as we present correlative evidence, we cannot exclude that effects that are correlated with baculum morphology but body, testes or seminal vesicle mass all did not influence paternity outcomes.

## Conclusions

In summary, we found that baculum shaft width influenced competitive fertilization success, but only for first-to-mate males. Furthermore, we found no clear evidence that this effect was caused by the baculum morphology enhancing sperm transport, the placement or removal of the copulatory plug or male copulatory behavior. We emphasize that the baculum might generally play a role in any of these. In addition, we encourage the use of both direct size measures and morphometric methods in research of the (house mouse) baculum, as our data suggest they might capture different aspects of the overall morphology. Finally, it remains unclear how universal the link of baculum morphology and fertilization success is in species that have bacula. Studies on the connection between male fitness and baculum morphology are still scarce (but see [23]). Phylogenetic studies suggest an important role of the baculum in sexual selection in rodents [21], but not in other mammalian orders [22], but see [58]. Overall, the evolutionary role of the baculum remains elusive in most species, and is potentially taxonomically diverse [12, 58]. Nevertheless, the association between baculum morphology and fertilization success seems well supported in house mice, and we hypothesize that this effect is rooted in the baculum’s stimulatory effects on the female during mating [13].

## Methods

### Sperm competition experiments

We made use of previously performed controlled mating trials described by Sutter and Lindholm [28, 39, 61] on laboratory-born F1–F3 descendants from a free-living population in Switzerland [45]. Briefly, these controlled mating trials were designed to induce competition between the ejaculates of two males. A virgin, receptive female was placed into a cage with one male, and the female was checked for a copulatory plug (indicating ejaculation; [62]) every 1–1.5 h (to avoid frequent interruptions that might disrupt normal behavior). If a copulatory plug was detected, it was either removed [28], or left intact. The female was then placed into a cage with a second male. This was a full-brother of the first male, reducing genetic or maternal variation between males [39]. Females were checked for new plugs every 30–60 min (as the timing of the second copulation is more predictable and occurs with little delay), and the second plug was removed if the first plug had also been removed. Copulatory plugs are routinely removed in house mouse sperm competition experiments [32, 43], with no negative impacts on the resumption of copulation with a subsequent male, pregnancy rates or litter size [28, 63]. For all brother pairs, trials were repeated until both treatments (plug removed/not removed) had resulted in pregnancy (with three exceptions), determined nine days *post coitum*. Males were sexually rested for a minimum of three days between trials. Females were sacrificed 9 days *post coitum* and embryos were removed and genotyped at 12 microsatellite markers to determine paternity [39]. For paternity data, here we only used trials where the competing males had the same *t* genotype (+ /*t* or + / +), because in + /*t* vs + / + competition genotype is a very strong determinant of fertilization success [39]. To test the third hypothesis concerning only baculum morphology and copulatory behavior, we extended the dataset by including competing males of different genotypes (i.e., + / + vs + /*t*).

Copulatory behavior was quantified using video recordings of the trials [38] and the following behavioral measures were documented: the number of copulatory bouts (mounts and mounts with intromissions) until ejaculation, the mean duration of all copulatory bouts, the latency to ejaculation (from the first mount) and the total duration of genital contact during ejaculation [38, 61]. Trials without ejaculation by the second male (12 out of 64 trials) were excluded for paternity analysis but included for behavioral analyses. To minimize observer bias, here and in the following blinded methods were used.

### Baculum measurements (detailed methods in supplementary material)

**Fig. 3:**
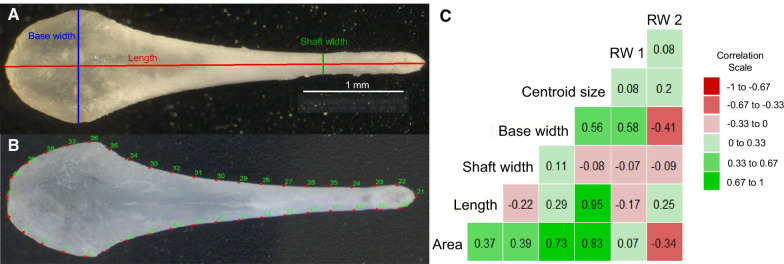
**A** Baculum with measurements for length, base (maximal) and shaft (minimal) width. **B** Baculum with numbered landmarks (red dots). Landmark 1 and 21 are fixed landmarks and all other landmarks are semi-sliding. **C** Correlation plot of all baculum measures

After euthanasia of the male by gradual CO_2_ filling in their home cage, the penis was immediately dissected out and the bone was cleaned (modified protocol after [24, 32, 33]). Bacula were photographed at 45× magnification through a microscope alongside a micrometer scale for size calibration (Fig. 3A). All pictures and measurements were taken blindly with respect to male ID and treatment by the first author. Repeatability was measured by intra-class correlation coefficients (ICC; see Additional file 1: Table S2) using R package ‘ICC’ [64]. We refer to the direct measurements (area, width and length) as ‘size’ and to the geometric morphometrics parameters as ‘shape’; we appreciate that neither of them purely reflects size or shape. For example, direct measurements are influenced by shape, and the measure of centroid size in the geometric morphometrics analysis is a measure of size.

i) Baculum size: direct measurements
  The bacula were measured using ImageJ (v1.49 [65]; after size calibration. The total length, base and shaft width were measured on a straight line by hand (Fig. 3A). Base width represents the width at the widest part of the base of the baculum, and shaft width the width at the narrowest part of the shaft (Fig. 3A).
ii) Baculum shape: geometric morphometrics
  In addition to the direct measurements of the baculum, its shape was quantified morphometrically using *tpsDig2* (v2.31; [66]). Two fixed and 38 semi-sliding landmarks were used to outline the baculum (modified after [31, 32]). Fixed landmarks were placed on the most proximal and distal positions of the baculum (Fig. 3B; Additional file 1: Figure S1). Analysis files were created with the pictures for measuring and appending curves to landmarks using *tpsUtil* (v1.76; [66]). Centroid size and relative warps were extracted using *tpsRelw* (v1.69; [66]). Centroid size is a measure that represents size independently from shape in the absence of allometry (i.e., centroid size is only correlated with shape if they change together; [67]). The relative warps (RW) express the variation of shape relative to the consensus configuration across all bacula [67]. The repeatability of geometric morphometric measurements of relative warp scores was analyzed by re-landmarking the same set of pictures twice (Additional file 1: Table S2).

Shape description of RW scores (i.e., what kind of alteration in shape is described by the RW) was assessed by plotting the extremes and vector-plots of the RW using *tpsRelw* (v1.69 [66]; Additional file 1: Figures S2 and S3). The first two relative warps are presented in the subsequent analyses. These explain together over 60% of the variation in shape (Additional file 1: Table S3). Lower scores for relative warp score 1 (RW1) indicate that the baculum has a ‘stretched’ base (and dorsal base-end) while higher scores mean a longitudinally ‘compressed’ base (and dorsal base-end; Additional file 1: Figure S2). Thus, the base is relatively wider but relatively shorter for larger RW1 values and narrower but relatively longer for negative values of RW1. Larger RW1 scores also describe relatively shorter bacula, due to the ‘compressed’ base. Relative warp score 2 describes variation in the width of the base of the baculum. Negative scores mean a relatively wider base, while positive scores mean a relatively narrower base (Additional file 1: Figure S3). Larger RW2 scores also describe a slight increase in relative baculum length.

### Statistical analyses

We analyzed baculum size (area, length and width) and shape (centroid size and relative warps 1 and 2) separately to assess if both measuring techniques lead to the same conclusions. All baculum measurements as well as body mass were standardized to a mean of 0 and a standard deviation of 1 to improve model conversion.

We analyzed the proportion of embryos sired by the first-to-mate male in binomial generalized linear mixed models (GLMMs) with a beta-binomial family using the package ‘glmmTMB’ [68] in R [69, 70]. We included baculum measurements as predictors and male ID as a random factor, and weighted samples by litter size. Overall, 48 different males were used in the mating trials, but most were tested multiple times. We included body mass and the genotype (+ / + or + /*t*) as covariates, and subsequently discarded variables with a variance inflation factor larger than 3 (Additional file 1: Table S4), resulting in the omission of baculum area from the models. We used full models to reduce the number of tests run and to avoid the “winner’s curse” (i.e., overestimated effect sizes leading to false-positives; [46, 47]), and adjusted p-values for multiple testing using false discovery rates [71]. In addition, we also applied a model selection approach, using AICc values [72] using the ‘MuMIn’ package [73]; data not shown. The results of these two approaches led to similar conclusions, except where stated otherwise.

First, we tested the relationship between baculum morphology and fertilization success (see Table 1). Here we included separate predictors about baculum morphology of the first- and second-to-mate males in the same model. We included genotype (+ / + vs + / + or + /*t* vs + /*t*) and the treatment of removing the copulatory plug in the model, as both are known to influence fertilization outcomes [38]. To address our questions about whether baculum morphology influences (1) sperm transport and (2) copulatory plug deposition/removal, we also included interactions between baculum morphology and (1) *t* genotype (+ / + or + /*t*) (see Additional file 1: Tables S5–S8) or (2) experimental removal of the plug (see Tables 2 and Additional file 1: Tables S9–S11). Because of the lowered statistical power due to interactions in the model, we tested first-to-mate and second-to-mate males in separate models.

In addition, we wanted to explore if baculum size or shape covaried with male copulatory behavior, to test for a potential role in vaginal stimulation (Additional file 1: Tables S13–S16). Here we used an extended dataset (for details see “Methods”—Sperm competition experiments) with 72 trials, 15 of which included competing males of different genotype (+ / + vs + /*t*). As males were tested repeatedly, we had baculum measurements from 32 first-to-mate and 32 s-to-mate males. We used full models separately for each of the different behavioral measures, focusing on four parameters of copulatory behavior that might covary with baculum morphology: the number of copulatory bouts, average bout duration, ejaculation latency (time between the first bout and ejaculation) and total time in copula. Data was log_10_ or sqrt transformed if appropriate to improve normality of model residuals (for details see model tables). We separately tested baculum size and shape measurements, and first- and second-to-mate males. We included body mass as a covariate in all models, but did not include *t* genotype, as + / + and + /*t* males do not differ in copulatory behavior [38].

## Supplementary Information

**Additional file 1.** Electronic Supplementary Material.

**Additional file 2.** Raw Data.

## Data Availability

All data generated or analysed during this study are included in Additional file 2.
