# Supplementary material for: The baculum affects paternity success of first but not second males in house mouse sperm competition
Source: BMC Ecol Evol. 2021 Aug 12;21:159. doi: 10.1186/s12862-021-01887-6 (PMC8359600; doi:10.1186/s12862-021-01887-6)
Supplement: Supplementary file 1 — Additional file 1. Electronic Supplementary Material. [file 12862_2021_1887_MOESM1_ESM.pdf]

Electronic Supplementary Material

The baculum affects paternity success of first but not second males in house mouse sperm competition

Contents

1. Detailed baculum measurement protocol 2
1.1. Baculum measurements 2
1.2. Baculum size: Direct measurements 2
1.3. Baculum shape: Geometric morphometrics 2
2. Baculum measurements: variation, repeatability, variance inflation factors 3
3. Fitness effects: Interactions between baculum morphology and genotype 6
3.1. First male – Size 6
3.2. First male – Morphometrics 7
3.3. Second male – Size 7
3.4. Second male – Morphometrics 8
4. Fitness effects: Interactions between baculum morphology and plug removal 8
4.1. First male – Size 8
4.2. First male – Morphometrics 9
4.3. Second male – Size 9
4.4. Second male – Morphometrics 10
5. Copulatory behaviour and the baculum 10
5.1. Morphometrics for larger dataset 10
5.2. First male 12
5.3. Second male 14
6. No morphological differences between +/+ and +/t. 16
7. No influence of testes and seminal vesicle weight. 16
8. Model selection via AICc. 17
8.1. Genotype interaction: Second male – Morphometrics 17
9. References 18

1. Detailed baculum measurement protocol

1.1. Baculum measurements

After euthanasia of the male, the penis was immediately dissected out. For baculum preparation, most penis tissue was carefully removed using dissection tools (partially under a dissection microscope). The remaining tissue was soaked in an Eppendorf tube in 1 ml of 0.05 g/ml KOH for 19-22h, after which the small distal tip of the bone and all remaining tissue was removed (modified after Ramm et al. 2010; Stockley et al. 2013; Simmons and Firman 2014). The bacula were then stored at 4°C in Eppendorf tubes containing 1ml of 100% Ethanol. Bacula were photographed through a microscope (Nikon SMZ 745T; Nikon GmbH, Düsseldorf, Germany) against a black background, alongside a micrometer scale for size calibration (Fig 1A). Photos were taken at 45x magnification with a Basler acA1920-40uc camera and the program Pylon Viewer (Basler AG; Basler AG, Ahrensburg, Germany). Each baculum was photographed from both sides. The side with a rather concave baculum base was defined 'upper' side and the rather convex side 'down' side. Repeatability was assessed comparing 'upper' and 'down' side measurements. All pictures and measurements were taken blindly with respect to male ID and treatment by the first author. Repeatability was measured by intra-class correlation coefficients (ICC; see Table S2) using R package 'ICC' (Wolak et al. 2012). We refer to the direct measurements (area, width and length) as 'size' and to the geometric morphometrics parameters as 'shape'; we appreciate that neither of them purely reflects size or shape. For example, direct measurements are influenced by shape, and the measure of centroid size in the geometric morphometrics analysis is a measure of size.

1.2. Baculum size: Direct measurements

The bacula were measured using ImageJ (v1.49; Schneider et al. 2012) after size calibration. The picture was first transformed to black and white applying a threshold. After transformation, the area was measured automatically using the 'wand' tool provided by ImageJ. The threshold was set manually for each image due to slightly varying light conditions and baculum brightness, but there was no correlation between threshold and measured baculum area (data not shown). All other measures were taken on the original picture. The total length, base and shaft width were measured on a straight line by hand (Figure 1A). Base width represents the width at the widest part of the base of the baculum, and shaft width the width at the narrowest part of the shaft (Figure 1A). Baculum measurements were repeatable, with ICC larger than 0.68. Paired t-tests were used to compare both measures (Table S1). The sides were not significantly different (not shown) and the means of both measures were used in all subsequent analyses concerning size measurements.

1.3. Baculum shape: Geometric morphometrics

In addition to the direct measurements of the baculum extracted using *ImageJ*, its shape was quantified morphometrically using *tpsDig2* (v2.31; Rohlf 2015). Two fixed and 38 semi-sliding landmarks were used to outline the baculum (modified after Simmons and Firman 2014; André et al. 2018). Fixed landmarks were placed on the most proximal and distal positions of the baculum (Figure 1B; Figure S1). The 38 semi-sliding landmarks were equally spaced on the periphery of the baculum and set as sliding (i.e. moving between the neighboring landmarks) in the analysis program. Analysis files were created with the pictures for measuring and appending curves to landmarks using *tpsUtil* (v1.76; Rohlf 2015). Centroid size and relative warps were extracted using *tpsRelw* (v1.69; Rohlf 2015). Centroid size is a measure that represents size independently from shape in the absence of allometry (i.e. centroid size is only correlated with shape if they change together; Zelditch et al. 2004). The relative warps (RW) express the variation of shape relative to the consensus configuration across all

bacula (Zelditch et al. 2004). 'Upper' and 'down' sides of the baculum were analyzed separately, because RW appeared to be different for the sides and the flipped orientation of the baculum might influence shape measures. Nevertheless, because results for 'upper' and 'down' side were qualitatively (and often also quantitatively) similar, only the results for the 'upper' side are reported for brevity. Additionally, the repeatabilities for centroid size and relative warp scores were analyzed by re-measuring the same set of pictures twice (Table S2).

Shape description of RW scores (i.e. what kind of alteration in shape is described by the RW) was assessed by plotting the extremes and vector-plots of the RW using *tpsRelw* (v1.69; Rohlf 2015; Figures S2 & S3). The first two relative warps are presented in the subsequent analyses. These explain together over 60% of the variation in shape (Table S3). Lower scores for relative warp score 1 (RW1) indicate that the baculum has a 'stretched' base (and dorsal base-end) while higher scores mean a longitudinally 'compressed' base (and dorsal base-end; Figure S2). Thus, the base is relatively wider but shorter for larger RW1 values and narrower but longer for negative values of RW1. Larger RW1 scores also describe shorter bacula, due to the 'compressed' base. Relative warp score 2 describes variation in the width of the base of the baculum. Negative scores mean a wider base, while positive scores mean a narrower base (Figure S3). Larger RW2 scores also describe slightly longer bacula.

2. Baculum measurements: variation, repeatability, variance inflation factors

The following section of figures presents what kind of shape variation the relative warp scores describe. For the different relative warps, we present the consensus configuration, negative/positive approximate minimum/maximum of warp scores and an extreme of negative/positive 0.1.

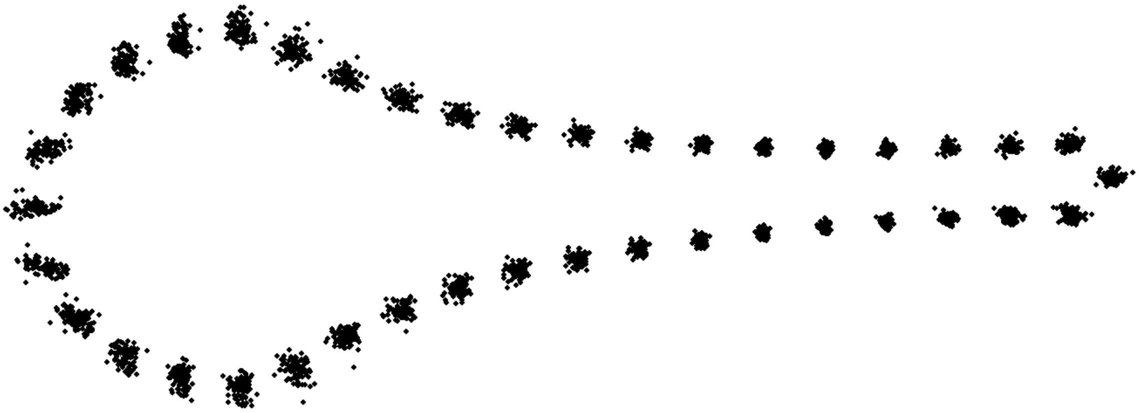

Figure S1: Plot of all individual landmark positions of all baculum samples. 40 landmarks with two fixed and 38 semi-sliding.

Relative warp score 1 mainly describes variation in the base of the baculum, meaning that the baculum has a "stretched" base (and dorsal base-end) for lower scores and a "compressed" base (and dorsal base-end) for higher scores (see figures S2). The base is wider for high RW1 values and narrower, while longer, for negative values of RW 1.

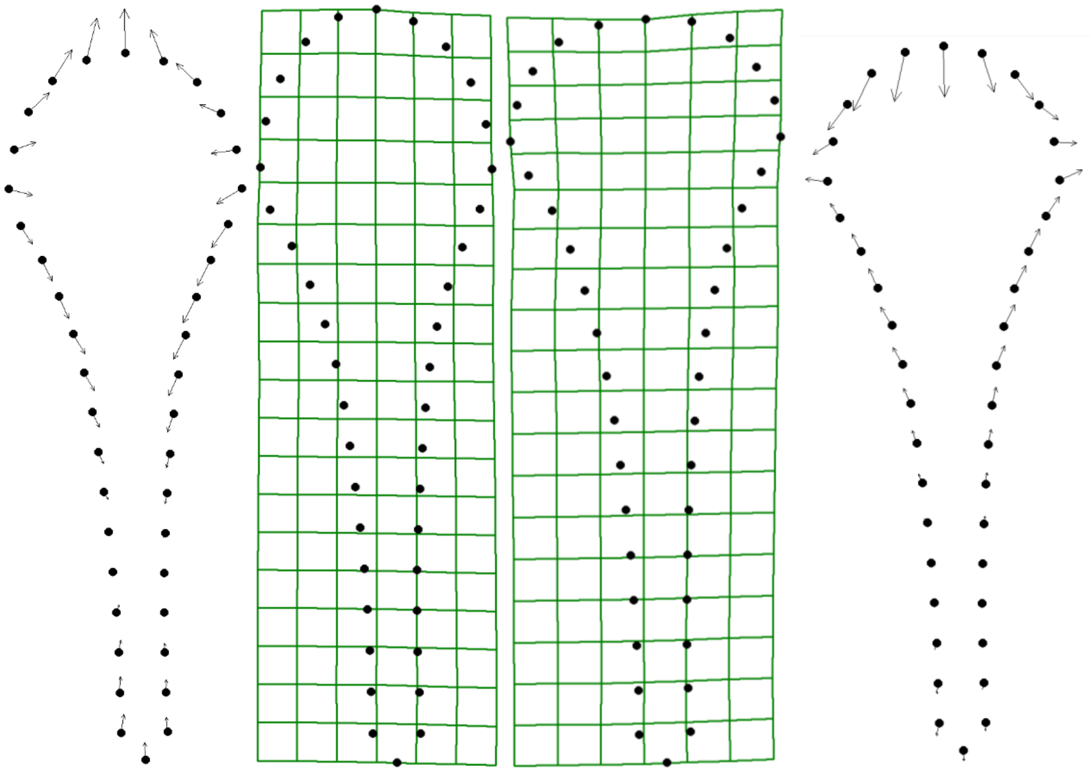

Figure S2: Relative warp score 1. Left to right RW: -0.1; -0.027; 0.041; 0.1. Change in RW represented by vectors for outer figures.

Relative warp score 2 describes variation in the width of the base of the bacula. Negative scores mean a wider base, while positive scores mean a narrower base (see figure S3). Large RW 2 scores also describe slightly longer bacula (and vice versa).

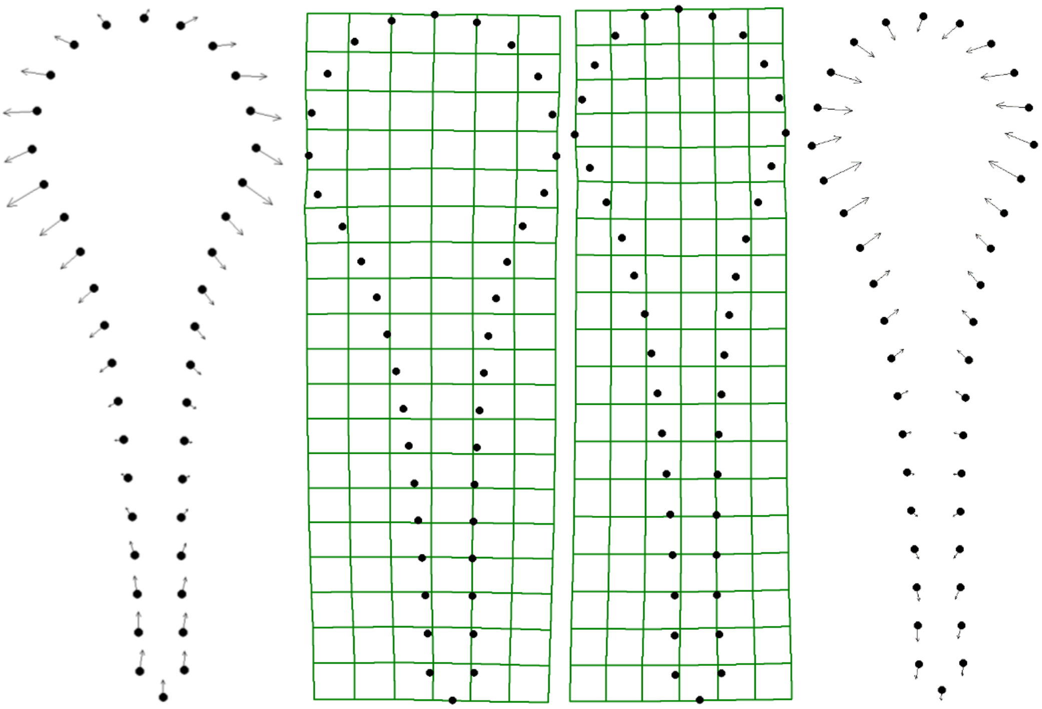

Figure S3: Relative warp score 2. Left to right RW: -0.1; -0.037; 0.029; 0.1. Change in RW represented by vectors for outer figures.

Table S1: Comparison of measurements form 'upper' and 'down' side of the baculum via two-sided, paired t-test. Given are t-value, degrees of freedom, p-value, 95% confidence interval (upper/lower level) and mean of the measurement ('upper' & 'down' side).

Measurement	t	df	p-value	ci (upper)	ci (lower)	Mean 'upper'	Mean 'down'
Area	-0.37	93.81	0.71	0.06	-0.09	2.83	2.85
Length	0.07	93.90	0.94	0.06	-0.06	4.30	4.29
Base width	0.43	94	0.66	0.03	-0.02	1.44	1.43
Shaft width	-1.51	91.06	0.13	0.002	-0.01	0.27	0.28
Centroid size	0.28	91.99	0.78	0.13	-0.10	8.76	8.74
Relative warp 1	0.16	92	0.87	0.007	-0.006	0.004	0.003
Relative warp 2	0.23	91.97	0.82	0.005	-0.004	0.001	0.0007

Table S2: Interclass correlation coefficients (ICC) of baculum measurements, with lower/upper confidence interval, N (sample size), number of measurements per individual (if unbalanced calculated after Lessells & Boag (1987), the within individual variance and among individual variance.

Measurement	ICC	Lower CI	Upper CI	n	k	Var. within	Var. among
Area	0.98	0.96	0.99	48	2	0.0007	0.03
Length	0.99	0.98	0.99	48	2	0.0002	0.02
Base width	0.99	0.98	0.99	48	2	0.00004	0.004
Shaft width	0.68	0.50	0.81	48	2	0.0001	0.0003
Centroid size	0.99	0.98	0.99	47	2	0.0007	0.08
Relative warp 1	0.97	0.96	0.99	47	2	0.000006	0.0002
Relative warp 2	0.97	0.95	0.98	47	2	0.000003	0.0001

Table S3: Singular values and percentage of variance explained for the first two relative warps.

Measurement	Singular values	%	Cum %
Relative warp 1	0.16	39.42%	39.42%
Relative warp 2	0.11	21.34%	60.76%

Table S4: Variance inflation factors of (a) baculum size and (b) baculum shape models. Binomial GLMMs with OLRE. Model specified in left column. Additional rows refer to recalculated VIF after removals. NA means variable dropped due to variance inflation >3.

a)

Model	Area	Length	Shaft width	Base width	Weight	Genotype
Fitness and size	3.84	1.79	1.59	2.80	1.46	1.20
	NA	1.40	1.10	1.18	1.46	1.09
Fitness and shape	13.31	8.44	1.54	3.25	1.78	1.11
	NA	1.54	1.12	1.07	1.59	1.09

b)

Model	Centroid size	RW1	RW2	Weight	Genotype
Fitness and size	1.56	1.91	1.70	1.91	2.24
Fitness and shape	2.70	1.79	1.20	1.90	1.08

3. Fitness effects: Interactions between baculum morphology and genotype

3.1. First male – Size

Table S5: Results of beta-binomial GLMM for the proportion of embryos sired by the first male (weighted by litter size) including body mass and genotype as covariates. First male baculum size measures as predictors, including interactions with genotype. resid. df=30.

Variable	Estimate	Std. error	z value	p-value
Intercept	0.87	0.30	2.85	0.004
Length	-0.19	0.35	0.55	0.58
Shaft width	0.71	0.34	2.06	0.04
Base width	0.51	0.31	1.63	0.10
Interaction: Length x Genotype (+/*t*)	-0.05	0.61	0.09	0.93
Interaction: Shaft width x Genotype (+/*t*)	-0.45	0.75	0.60	0.55
Interaction: Base width x Genotype (+/*t*)	0.66	0.65	1.02	0.31
Body mass	0.29	0.34	0.85	0.39
Genotype (+/*t*)	-2.62	0.69	3.79	0.0001

3.2. First male – Morphometrics

Table S6: Results of beta-binomial GLMM for the proportion of embryos sired by the first male (weighted by litter size) including body mass and genotype as covariates. First male morphometric baculum measures as predictors including interactions with genotype. resid. df=28.

Variable	Estimate	Std. error	z value	p-value
Intercept	0.78	0.43	1.82	0.07
Centroid size	0.07	0.38	0.18	0.86
Relative warp 1	0.19	0.40	0.49	0.62
Relative warp 2	-0.39	0.54	0.73	0.47
Interaction: Centroid size x Genotype (+/*t*)	0.15	0.73	0.20	0.84
Interaction: Relative warp 1 x Genotype (+/*t*)	1.41	0.87	1.62	0.11
Interaction: Relative warp 2 x Genotype (+/*t*)	0.24	0.65	0.37	0.71
Body mass	0.13	0.37	0.35	0.72
Genotype (+/*t*)	-3.59	1.14	3.15	0.002

3.3. Second male – Size

Table S7: Results of beta-binomial GLMM for the proportion of embryos sired by the second male (weighted by litter size) including body mass and genotype as covariates. Second male baculum size measures as predictors, including interactions with genotype. resid. df=31.

Variable	Estimate	Std. error	z value	p-value
Intercept	-0.99	0.33	3.01	0.003
Length	-0.13	0.40	0.33	0.74
Shaft width	-0.16	0.34	0.49	0.63
Base width	-0.04	0.39	-0.09	0.93
Interaction: Length x Genotype (+/*t*)	0.45	0.59	0.76	0.44
Interaction: Shaft width x Genotype (+/*t*)	0.003	0.60	0.005	0.99
Interaction: Base width x Genotype (+/*t*)	-0.27	0.53	-0.51	0.61
Body mass	-0.46	0.29	-1.59	0.11
Genotype (+/*t*)	2.24	0.54	4.11	0.00004

3.4. Second male – Morphometrics

Table S8: Results of beta-binomial GLMM for the proportion of embryos sired by the second male (weighted by litter size) including body mass and genotype as covariates. Second male morphometric baculum measures as predictors, including interactions with genotype. resid. df=31.

Variable	Estimate	Std. error	z value	p-value
Intercept	-1.02	0.34	3.04	0.002
Centroid size	-0.08	0.46	0.17	0.87
Relative warp 1	0.26	0.37	0.69	0.49
Relative warp 2	-0.12	0.40	0.29	0.77
Interaction: Centroid size x Genotype (+/*t*)	-0.50	0.91	0.55	0.58
Interaction: Relative warp 1 x Genotype (+/*t*)	-0.86	0.79	1.09	0.28
Interaction: Relative warp 2 x Genotype (+/*t*)	0.66	0.56	1.18	0.24
Body mass	-0.40	0.33	1.23	0.22
Genotype (+/*t*)	2.36	0.60	3.93	0.00008

4. Fitness effects: Interactions between baculum morphology and plug removal

4.1. First male – Size

Table S9: Results of beta-binomial GLMM for the proportion of embryos sired by the first male (weighted by litter size) including body mass and genotype as covariates. First male baculum size measures as predictors, including interactions with plug removal treatment. resid. df=29.

Variable	Estimate	Std. error	z value	p-value
Intercept	1.42	0.44	3.21	0.001
Length	0.17	0.54	0.31	0.75
Shaft width	0.99	0.51	1.96	0.05
Base width	0.38	0.32	1.20	0.23
Interaction: Length x Plug removal	-0.43	0.61	0.70	0.48
Interaction: Shaft width x Plug removal	-0.09	0.62	-0.15	0.88
Interaction: Base width x Plug removal	0.53	0.51	1.04	0.30
Body mass	0.30	0.32	0.94	0.35
Genotype (+/*t*)	-2.23	0.56	3.97	0.00007
Plug removal	-1.01	0.53	-1.91	0.06

4.2. First male – Morphometrics

Table S10: Results of beta-binomial GLMM for the proportion of embryos sired by the first-to-mate male (weighted by litter size) including body mass and genotype as covariates. First male morphometric baculum measures as predictors, including interactions with plug removal treatment. resid. df=27.

Variable	Estimate	Std. error	z value	p-value	p-adj
Intercept	1.20	0.53	2.27	0.02	0.08
Centroid size	0.33	0.43	0.78	0.44	0.73
Relative warp 1	0.52	0.42	1.22	0.22	0.55
Relative warp 2	0.23	0.39	0.60	0.55	0.78
Interaction: Centroid size x Plug removal	0.07	0.59	0.11	0.91	0.91
Interaction: Relative warp 1 x Plug removal	-0.14	0.66	0.21	0.83	0.91
Interaction: Relative warp 2 x Plug removal	-2.28	0.98	2.32	0.02	0.08
Body mass	-0.15	0.41	0.36	0.72	0.89
Genotype (+/*t*)	-2.67	1.02	2.61	0.009	0.08
Plug removal	-0.52	0.59	0.87	0.38	0.73

4.3. Second male – Size

Table S11: Results of beta-binomial GLMM for the proportion of embryos sired by the second male (weighted by litter size) including body mass and genotype as covariates. Second male baculum size measures as predictors, including interactions with plug removal treatment. resid. df=30.

Variable	Estimate	Std. error	z value	p-value
Intercept	-1.29	0.48	2.65	0.008
Length	0.17	0.42	0.40	0.69
Shaft width	-0.45	0.45	1.02	0.31
Base width	-0.20	0.36	0.56	0.58
Interaction: Length x Plug removal	-0.15	0.53	0.29	0.77
Interaction: Shaft width x Plug removal	0.48	0.58	0.82	0.41
Interaction: Base width x Plug removal	-0.15	0.52	0.29	0.77
Body mass	-0.43	0.31	1.40	0.16
Genotype (+/*t*)	2.34	0.64	3.66	0.0003
Plug removal	0.47	0.53	0.89	0.37

4.4. Second male – Morphometrics

See table 2 in main text.

5. Copulatory behaviour and the baculum

5.1. Morphometrics for larger dataset

Table S12: Singular values and percent explained for relative warps of larger dataset for behaviour analysis.

Measurement	Singular values	%	Cum %
Relative warp 1	0.18	38.73%	38.73%
Relative warp 2	0.14	21.70%	60.42%
Relative warp 3	0.10	11.54%	71.97%

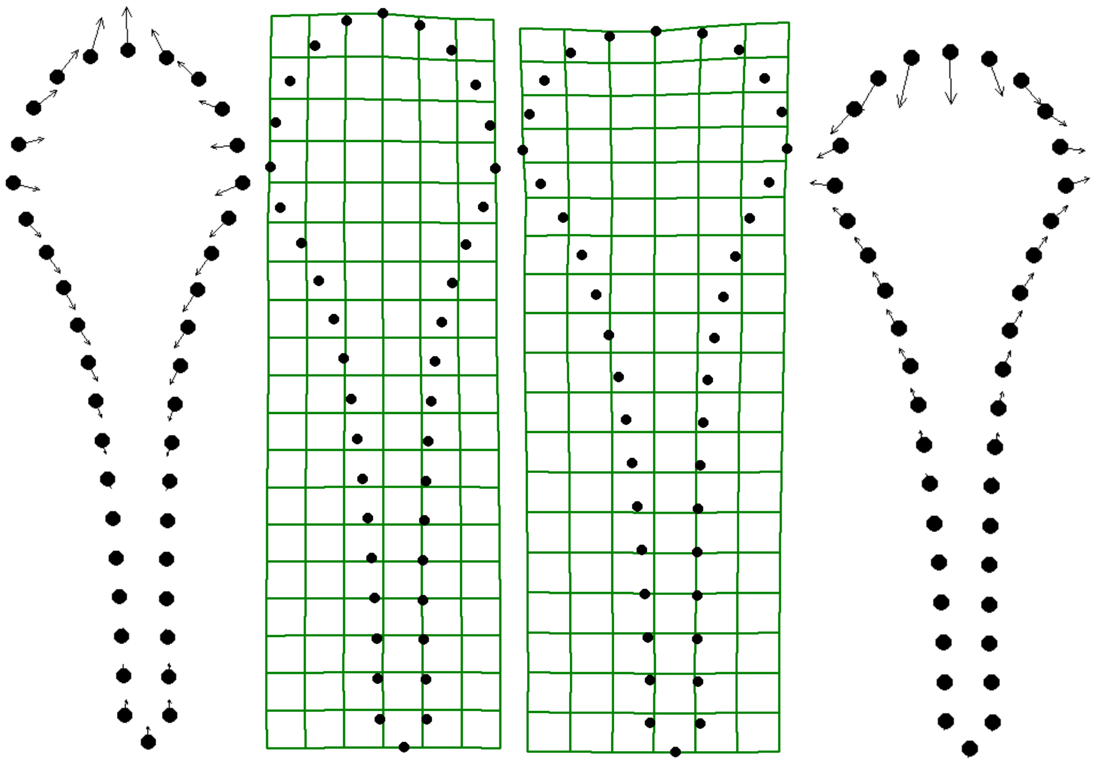

Figure S4: Relative warp score 1 of larger dataset for behaviour analysis. Left to right RW: -0.1; -0.027; 0.039; 0.1. Change in RW represented by vectors for outer figures.

Relative warp score 1 means that the baculum has a "stretched" base (and dorsal base-end) for lower scores and a "compressed" base (and dorsal base-end) for higher scores (see figures S5). The base is wider for high RW1 values and narrower, while longer, for negative values of RW 1.

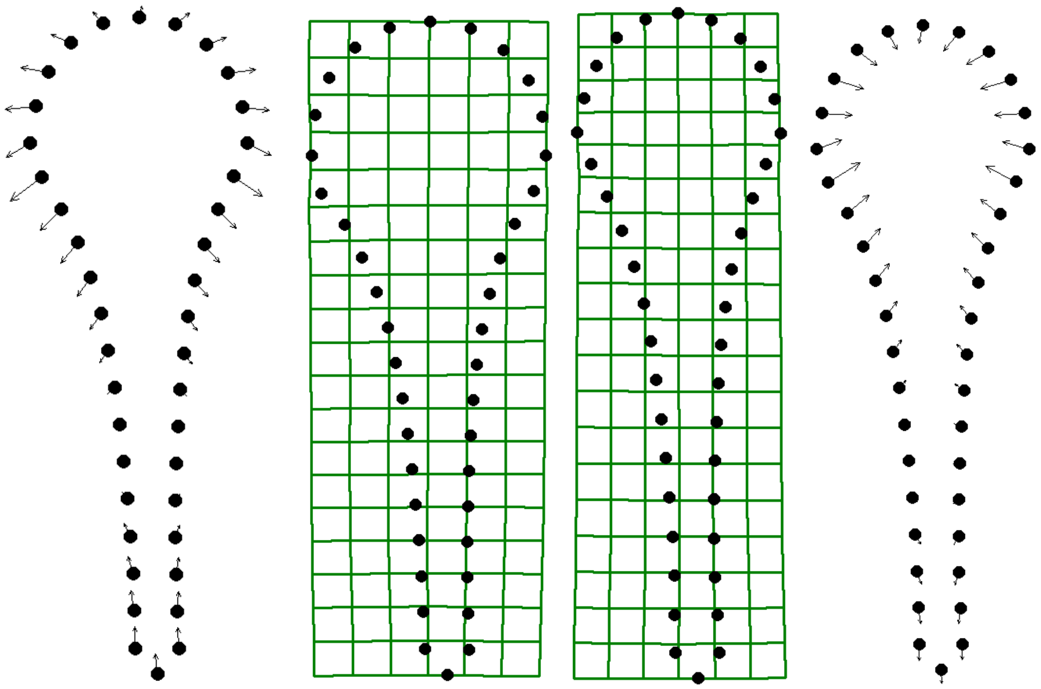

Figure S5: Relative warp score 2 of larger dataset for behaviour analysis. Left to right RW: -0.1; -0.035; 0.030; 0.1. Change in RW represented by vectors for outer figures.

Relative warp score 2 describes variation in the wideness of the base of the bacula. Negative scores mean a wider base, while positive scores mean a smaller base (see figure S6). Large RW 2 scores also describe slightly longer bacula.

5.2. First male

Table S13: Results of linear mixed model for behavioural measurements (specified in 'model') by the first male including body mass as covariate. Size baculum measures as predictors.

Model	Variable	Estimate	Std. error	df	t value	p-value
log_{10} Copulatory bouts	Intercept	8.48	3.17	27.32	2.67	0.01
	log_{10} Length	-4.88	4.37	31.12	-1.12	0.27
	log_{10} Shaft width	1.99	1.60	26.30	1.24	0.23
	log_{10} Base width	1.84	1.57	26.01	1.17	0.25
	log_{10} Body mass	-2.52	2.11	32.28	-1.20	0.24
log_{10} Average bout duration [s]	Intercept	1.52	1.33	66.00	1.14	0.26
	log_{10} Length	3.79	1.84	66.00	2.06	0.04
	log_{10} Shaft width	0.81	0.67	66.00	1.20	0.23
	log_{10} Base width	0.69	0.66	66.00	1.05	0.30
	log_{10} Body mass	-1.68	0.89	66.00	-1.89	0.06
log_{10} In copula duration [min]	Intercept	1.59	0.99	26.66	1.60	0.12
	log_{10} Length	0.02	1.36	28.76	0.01	0.99
	log_{10} Shaft width	0.04	0.50	25.25	0.08	0.94
	log_{10} Base width	0.03	0.49	25.18	0.05	0.96
	log_{10} Body mass	-0.29	0.65	33.20	-0.45	0.66

Table S14: Results of linear mixed model for behavioural measurements (specified in ‘model’) by the first male including body mass as covariates. Morphometric baculum measures as predictors.

Model	Variable	Estimate	Std. error	df	t value	p-value	p-adj
Copulatory bouts	Intercept	8.72	4.41	27.62	1.98	0.06	-
	log_{10} Centroid size	-5.95	4.83	26.51	-1.23	0.23	-
	Relative warp 1	1.40	3.58	28.72	0.39	0.70	-
	Relative warp 2	-7.55	6.21	33.98	-1.22	0.23	-
	log_{10} Body mass	-1.47	2.30	35.21	-0.64	0.53	-
log_{10} Average bout duration [s]	Intercept	1.21	1.86	63.00	0.65	0.52	-
	log_{10} Centroid size	3.70	2.04	63.00	1.81	0.07	-
	Relative warp 1	1.48	1.52	63.00	0.97	0.33	-
	Relative warp 2	2.37	2.65	63.00	0.90	0.37	-
	log_{10} Body mass	-2.47	0.98	63.00	-2.52	0.01	-
Sqrt Ejaculation latency [min]	Intercept	979.76	371.43	63.00	2.64	0.01	0.05
	log_{10} Centroid size	-814.13	406.12	63.00	-2.00	0.05	0.12
	Relative warp 1	270.23	302.55	63.00	0.89	0.37	0.62
	Relative warp 2	-20.83	528.34	63.00	-0.04	0.97	0.97
	log_{10} Body mass	-104.46	195.87	63.00	-0.53	0.60	0.74
log_{10} In copula duration [min]	Intercept	1.14	1.32	27.33	0.86	0.40	-
	log_{10} Centroid size	1.04	1.45	26.20	0.72	0.48	-
	Relative warp 1	1.80	1.07	28.46	1.68	0.10	-
	Relative warp 2	0.46	1.86	33.72	0.25	0.81	-
	log_{10} Body mass	-0.67	0.69	34.85	-0.97	0.34	-

5.3. Second male

Table S15: Results of linear mixed model for behavioural measurements (specified in 'model') by the second male including body mass and as covariates. Size baculum measures as predictors.

Model	Variable	Estimate	Std. error	df	t value	p-value
Copulatory bouts	Intercept	34.93	105.01	63.00	0.33	0.74
	$\log_{10}$ Length	-216.26	159.09	63.00	-1.36	0.18
	$\log_{10}$ Shaft width	78.74	63.41	63.00	1.24	0.22
	$\log_{10}$ Base width	67.23	64.35	63.00	1.04	0.30
	$\log_{10}$ Body mass	120.57	70.41	63.00	1.71	0.09
$\log_{10}$ Average bout duration [s]	Intercept	1.57	1.04	63.00	1.51	0.13
	$\log_{10}$ Length	2.44	1.57	63.00	1.56	0.12
	$\log_{10}$ Shaft width	-0.89	0.63	63.00	-1.42	0.16
	$\log_{10}$ Base width	-0.78	0.63	63.00	-1.22	0.23
	$\log_{10}$ Body mass	-1.79	0.69	63.00	-2.57	0.01
Sqrt Ejaculation latency [min]	Intercept	-91.87	93.46	23.94	-0.98	0.33
	$\log_{10}$ Length	-166.08	141.18	25.58	-1.18	0.25
	$\log_{10}$ Shaft width	-37.19	56.99	19.87	-0.65	0.52
	$\log_{10}$ Base width	-37.03	57.96	19.23	-0.64	0.53
	$\log_{10}$ Body mass	167.99	61.71	34.75	2.72	0.01
$\log_{10}$ In copula duration [min]	Intercept	1.10	0.96	29.40	1.14	0.26
	$\log_{10}$ Length	-2.43	1.44	31.15	-1.68	0.10
	$\log_{10}$ Shaft width	-0.58	0.60	26.33	-0.97	0.34
	$\log_{10}$ Base width	-0.53	0.61	26.23	-0.87	0.39
	$\log_{10}$ Body mass	0.88	0.60	46.80	1.47	0.15

Table S16: Results of linear mixed model for behavioural measurements (specified in 'model') by the second male including body mass as covariates. Morphometric baculum measures as predictors.

Model	Variable	Estimate	Std. error	Df	t value	p-value
Copulatory bouts	Intercept	-64.63	143.37	63.00	-0.45	0.65
	log_{10} Centroid size	-33.85	190.32	63.00	-0.178	0.86
	Relative warp 1	122.23	164.56	63.00	0.743	0.46
	Relative warp 2	-303.12	164.65	63.00	-1.841	0.07
	log_{10} Body mass	94.43	75.18	63.00	1.256	0.21
log_{10} Average bout duration [s]	Intercept	2.18	1.43	63.00	1.53	0.13
	log_{10} Centroid size	1.12	1.90	63.00	0.59	0.56
	Relative warp 1	-0.80	1.64	63.00	-0.49	0.63
	Relative warp 2	2.71	1.64	63.00	1.65	0.10
	log_{10} Body mass	-1.63	0.75	63.00	-2.17	0.03
Sqrt Ejaculation latency [min]	Intercept	-31.09	129.57	14.84	-0.24	0.81
	log_{10} Centroid size	-156.21	171.10	16.63	-0.91	0.37
	Relative warp 1	89.89	148.57	15.37	0.60	0.55
	Relative warp 2	-13.41	147.93	16.39	-0.09	0.93
	log_{10} Body mass	165.02	65.44	32.56	2.52	0.02
log_{10} In copula duration [min]	Intercept	1.96	1.43	23.42	1.37	0.18
	log_{10} Centroid size	-2.31	1.85	27.80	-1.25	0.22
	Relative warp 1	1.5	1.63	25.00	0.92	0.36
	Relative warp 2	0.17	1.62	24.66	0.11	0.92
	log_{10} Body mass	0.88	0.64	48.78	1.37	0.18

6. No morphological differences between +/+ and +/*t.*

Table S17: Unpaired, two sided 'Welch Two Sample' t-tests for differences between +/+ and +/*t* males in various morphological baculum features. p-value 'FDR' adjusted.

Measurement	t value	df	p-value	p adj.	Mean +/+	Mean +/*t*
Area	-1.31	20.11	0.20	0.29	8.69	8.81
Length	-0.78	19.42	0.44	0.44	4.27	4.31
Shaft width	1.43	21.88	0.17	0.29	0.28	0.27
Base width	-0.83	20.53	0.42	0.44	1.41	1.44
Centroid size	-1.31	20.11	0.20	0.29	8.69	8.81
Relative warp 1	-2.87	19.97	0.009	0.07	-0.006	0.009
Relative warp 2	-1.85	13.90	0.08	0.29	-0.001	0.006

7. No influence of testes and seminal vesicle weight.

Table S18: Results of generalised linear mixed model of family beta-binomial for the proportion of embryos sired by the first male including body mass, testes weight, seminal vesicle weight and genotype as covariates. Model weight by the total number of embryos per female. df=36.

Variable	Estimate	Std. error	t value	p-value
Intercept	0.63	0.28	2.22	0.03
Shaft width	0.91	0.27	3.32	0.0009
Base width	0.39	0.22	1.80	0.07
Testes weight	-0.09	-0.05	1.67	0.09
Seminal vesicle weight	0.02	0.02	0.67	0.50
Body mass	0.19	0.25	0.76	0.44
Genotype (+/*t*)	-1.73	0.49	-3.55	0.0004

8. Model selection via AICc

Table S19: Results of generalized linear mixed models of family beta-binomial for the proportion of embryos sired by the first male (weighted by litter size) including body mass and genotype as covariates. Size and morphometric baculum measures in separate models as predictors. Models were compared by AICc, and the models with the lowest AICc are presented here (for full models see Table 1). M1/M2 = first/second-to-mate male.

Model	Variable	Estimate	Std. error	z value	p-value	p-adj
Baculum size	Intercept	0.93	0.32	2.96	0.003	0.006
(dimensions)	Shaft width M1	0.82	0.28	2.91	0.004	0.006
df= 33	Base width M1	0.49	0.24	2.06	0.04	0.04
	Body mass M2	0.60	0.26	2.30	0.02	0.03
	Genotype (+/*t*)	-2.38	0.56	4.28	<0.001	<0.001
Baculum shape (morphometrics)	Intercept	1.00	0.35	2.82	0.005	0.01
	Relative warp 1 M1	0.55	0.32	1.71	0.09	0.09
df= 32	Body mass M2	0.48	0.27	1.78	0.08	0.08
	Genotype (+/*t*)	-2.95	0.69	4.30	<0.001	<0.001

8.1. Genotype interaction: Second male – Morphometrics

Table S20: Results of generalized linear mixed model of family beta-binomial for the proportion of embryos sired by the second-to-mate male (weighted by litter size) including body mass and genotype as covariates. Morphometric baculum measures as predictors including plug removal treatment interactions. df=37. Models were compared by AICc, and the models with the lowest AICc are presented here (for full models see Table 2).

Variable	Estimate	Std. error	z value	p-value
Intercept	-0.99	0.35	2.80	0.005
Body mass	-0.48	0.26	1.87	0.06
Genotype (+/*t*)	2.44	0.62	3.92	0.00009

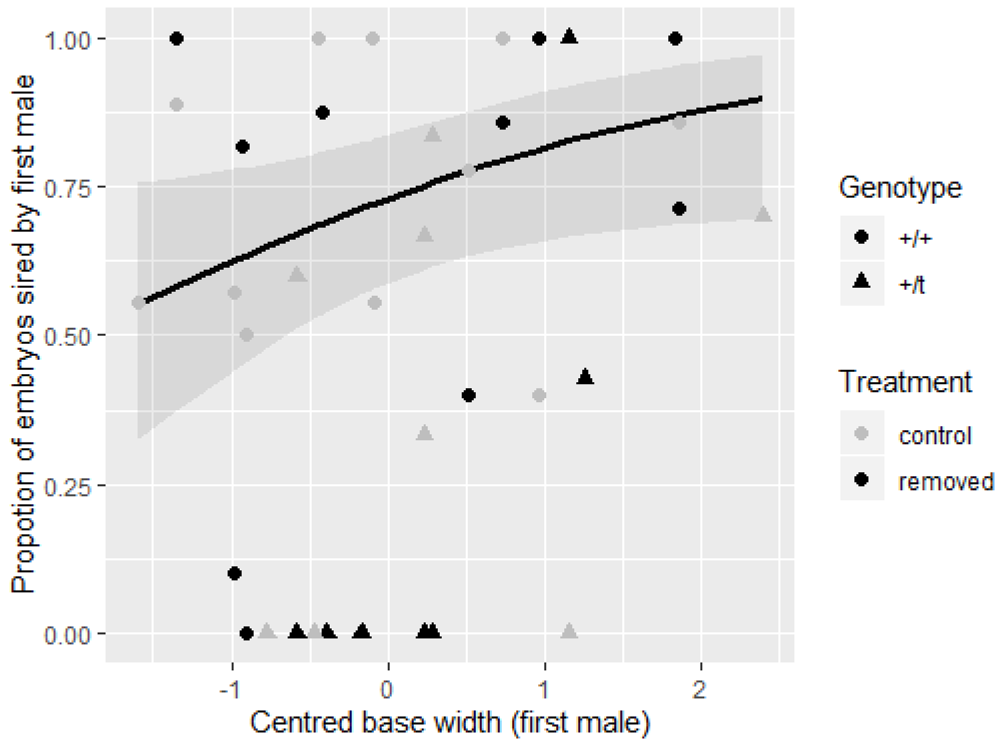

Figure S6: Proportion of embryos sired by the first male plotted against the centered and scaled base width of the first-to-mate male. Regression represents predicted values (with 95% confidence intervals in grey) from beta-binomial model.

9. References

André GI, Firman RC, & André GI, Firman RC, Simmons LW (2018) Phenotypic plasticity in genitalia: Baculum shape responds to sperm competition risk in house mice. Proc R Soc B Biol Sci. **285**:20181086.

Lessells CM, Boag PT (1987) Unrepeatable Repeatabilities: A Common Mistake. The Auk, 104(1):116–12.

Ramm SA, Khoo L, Stockley P (2010) Sexual selection and the rodent baculum: An intraspecific study in the house mouse (*Mus musculus domesticus*). Genetica. 138(1):129–137.

Rohlf FJ (2015) The tps series of software. Hystrix. 26(1):1–4.

Schneider CA, Rasband WS, Eliceiri KW (2012) NIH Image to ImageJ: 25 years of image analysis. Nature Methods 9:671-675.

Simmons LW, Firman RC (2014) Experimental evidence for the evolution of the mammalian baculum by sexual selection. Evolution. 68(1):276–283.

Stockley P, Ramm SA, Sherborne AL, Thom MDF, Paterson S, Hurst JL (2013) Baculum morphology predicts reproductive success of male house mice under sexual selection. BMC Biol. 11(1):66.

Wolak ME, Fairbairn DJ, Paulsen YR (2012) Guidelines for estimating repeatability. Methods Ecol Evol. 3(1):129–137.

Zelditch ML, Swiderski DL, Sheets HD, Fink WL (2004) Geometric morphometrics for biologists. Elsevier Inc.
